# Photocatalytic Overall Water Splitting by SrTiO_3_ with Surface Oxygen Vacancies

**DOI:** 10.3390/nano10122572

**Published:** 2020-12-21

**Authors:** Yanfei Fan, Yan Liu, Hongyu Cui, Wen Wang, Qiaoyan Shang, Xifeng Shi, Guanwei Cui, Bo Tang

**Affiliations:** College of Chemistry, Chemical Engineering and Materials Science, Collaborative Innovation Center of Functionalized Probes for Chemical Imaging in Universities of Shandong, Key Laboratory of Molecular and Nano Probes, Ministry of Education, Shandong Normal University, Jinan 250014, China; yffan810@163.com (Y.F.); ly2017020911@163.com (Y.L.); liuliucuicui123@163.com (H.C.); ww19862550729@163.com (W.W.); qiaoyanshang@sdnu.edu.cn (Q.S.); sxf0716@163.com (X.S.); tangb@sdnu.edu.cn (B.T.)

**Keywords:** photocatalysis, water splitting, strontium titanate, oxygen vacancy, hydrogen energy

## Abstract

Strontium Titanate has a typical perovskite structure with advantages of low cost and photochemical stability. However, the wide bandgap and rapid recombination of electrons and holes limited its application in photocatalysis. In this work, a SrTiO_3_ material with surface oxygen vacancies was synthesized via carbon reduction under a high temperature. It was successfully applied for photocatalytic overall water splitting to produce clean hydrogen energy under visible light irradiation without any sacrificial reagent for the first time. The photocatalytic overall water splitting ability of the as-prepared SrTiO_3_-C950 is attributed to the surface oxygen vacancies that can make suitable energy levels for visible light response, improving the separation and transfer efficiency of photogenerated carriers.

## 1. Introduction

Photocatalytic water splitting is a promising way to produce hydrogen clean energy [1,2]. Semiconductor materials have been widely used in this field because of their excellent photoelectric response characteristics. However, limited by the inappropriate redox potential, rapid recombination of photogenerated carriers or the backward reaction of H_2_ and O_2_ to H_2_O, most of them can only perform the semi-reaction of water splitting to produce hydrogen or oxygen alone assisted by sacrificial agents such as methanol, sodium sulfite or silver nitrate [3,4]. Therefore, although photocatalytic water splitting has been studied for several decades, there are few reported materials that can realize overall water splitting, which still remains a challenge in the photocatalytic research field [5,6,7,8].

Strontium Titanate (SrTiO_3_) has typical perovskite structure with the advantages of low cost and excellent chemical stability. It has been widely used as a photocatalyst [9,10]. However, due to the wide band gap, it can only utilize ultraviolet light (about 5% of sunlight) during photocatalytic reactions. Moreover, although its band structure suits the water redox potential levels [11], SrTiO_3_ powders alone cannot decompose water into H_2_ and O_2_ simultaneously because of rapid recombination of photogenerated carriers or fast backward reaction. It is necessary to modify the SrTiO_3_ particle to obtain an active photocatalyst for water splitting. Up to now, many outstanding methods, such as doped with metals or non-metals [12,13,14,15,16], or coupled with other semiconductors [17], have been investigated to solve the abovementioned issues. Recently, a modified aluminum-doped strontium titanate (SrTiO_3_: Al) photocatalyst was applied to perform overall water splitting with an external quantum efficiency of up to 96 per cent, which was still performed under UV light irradiation [18].

Oxygen vacancies on a particle surface often act as active sites and play an important role in photocatalytic reactions [19]. Moreover, they can provide trapping sites for photogenerated carriers and prevent them from rapid recombination. Most notably, they can build a surface energy state that can narrow the band gap, expanding the solar absorbance range to lower energy wavelengths [20]. Thus, rational control of oxygen vacancies on the catalyst surface is extremely important for improving the photocatalytic efficiency [21,22,23]. Although there are usually many oxygen vacancies in the inner part of bulk SrTiO_3_, they do not contribute to the sub-band gap photoexcitation or intrinsic magnetism of SrTiO_3_, and are different from the surface oxygen vacancies [20]. Therefore, it is necessary to synthesize SrTiO_3_ materials with abundant surface oxygen vacancies to realize overall photocatalytic water splitting under visible light conditions. Carbon material is a cheap and safe reductant that can capture oxygen atoms from the oxide surface to produce reductive species or create oxygen vacancies at a high temperature [24,25]. Herein, it was found that when the pristine SrTiO_3_ was heated under reducing atmosphere afforded by carbon, a disordered surface layer with amounts of oxygen vacancies was obtained. The as-prepared SrTiO_3_ with a grey color showed overall photocatalytic water splitting ability under visible light irradiation without a sacrifice reagent.

## 2. Materials and Methods

### 2.1. Materials

Ti(C_4_H_9_O)_4_,Sr(NO_3_)_2_, KOH, ammonia, NaClO_4_, terpinol, naphthol, polyvinyl alcohol (PVA), ethanol and active carbon power were purchased from Sinopharm Chemical Reagent Company. All the chemicals were of AR grade. Ultrapure water used in the experiment was Wahaha pure water.

### 2.2. Synthesis of SrTiO_3_-C950

SrTiO_3_ was synthesized by a hydrothermal method [26]. Typically, Ti(C_4_H_9_O)_4_ (2.72 g) and ethanol (70.00 mL) were mixed homogeneously by stirring at room temperature. Then, ammonia solution (2.50 mL of ammonia diluted with 30.00 mL of ethanol) was added to the mixed solution with stirring for one hour. The obtained precipitate was washed with distilled water several times and dispersed in 80.00 mL distilled water with vigorous stirring. Then, 5.08 g Sr(NO_3_)_2_, 2.24 g KOH, and 0.32 g polyvinyl alcohol (PVA) were added to the mixture solution. Finally, the obtained suspension was transferred into a stainless-steel Teflon-lined autoclave and heated at 180 °C for 12 h. The obtained solid product was washed with diluted nitric acid, distilled water and ethanol several times in turn and then dried at 80 °C for 4 h. Finally, the as-prepared SrTiO_3_ (0.50 g) and carbon (0.08 g) were well mixed. The mixture was calcined at 950 °C for 3 h with a heating rate of 5 °C/min under N_2_ atmosphere, and then naturally cooled at room temperature. The as-prepared sample was denoted as SrTiO_3_-C950. The mixture of SrTiO_3_ (0.50 g) and carbon (0.08 g) calcined at 300 °C for 3 h with a heating rate of 5 °C/min under N_2_ atmosphere, denoted as SrTiO_3_-C300, was used as reference samples.

### 2.3. Electrochemical Measurements

The photocurrent was measured by an electrochemical analyzer (CHI660D Instruments, Shanghai Chenhua Instrument Co., Ltd., Shanghai, China) with a standard three-electrode system. The electrolyte was a 0.10 M NaClO_4_ aqueous solution. The working electrode was prepared as follows. The slurry was obtained by grinding a mixture of the sample (0.05 g) and terpinol (0.10 g). Then, the slurry was coated onto a 4.00 cm × 1.00 cm indium tin oxide-coated glass (ITO glass) electrode by the doctor blade technique, dried in an oven, and calcined at 290 °C for 30 min under Ar conditions. The as-prepared sample, Pt sheet and saturated calomel electrode were used as the working electrode, counter electrode and reference electrode, respectively. Prior to the photocurrent measurements, the electrolyte (0.10 M NaClO_4_, pH = 6.56) was purged with Ar for 30 min.

The electrochemical impedance test was also carried out with a standard three-electrode system. The glassy carbon electrode deposited with the as-prepared samples was used as the working electrode. Pt sheet and saturated calomel electrode was used as the counter electrode and the reference electrode, respectively. The working electrode was prepared as follows: 4.00 mg the as-prepared sample was dispersed in 1.00 mL mixture of water and isopropanol with the volume ratio of 3:1, then 40.00 μL naphthol was added into the mixture and ultrasonicated for 30 min. The obtained slurry was dropped onto the glassy carbon electrode, and then dried naturally at room temperature. Finally, the prepared working electrode, counter electrode and reference electrode were immersed in a deionized water solution for testing.

### 2.4. Photocatalytic Water Splitting

The photocatalytic water splitting reaction was performed using an XPA-7 photocatalytic reaction instrument (Xujiang Electromechanical Plant, Nanjing, China). In a typical process, 0.40 g SrTiO_3_-C950, 0.20 g La_2_O_3_ and 10.00 mL water were mixed in a 20 mL quartz bottle at room temperature. The bottle was sealed with a silicone rubber septum. The suspension was thoroughly deaerated and purged with N_2_ for 30 min before the photocatalysis experiment. Then, the suspension was irradiated by a 1000 W Xe lamp with or without light cutoff filters (λ > 420 nm) under ambient conditions and magnetic stirring for a certain time. The gaseous production was analyzed by gas chromatography (FULI 9750, TCD, argon as the carrier gas, and 5 Å molecular sieve column).

The solar-to-hydrogen (STH) conversion efficiency was evaluated by using a 1000 W Xe lamp as the light source and 0.40 g catalyst in water. After irradiation for 48 h, an average of 360 µmol H_2_ was obtained, which corresponds to 93 J free energy. The incident light power was 162 mW/cm^2^, which was measured by a handheld Optical Power Meter (Newport 1916-R). The Light exposure area of the quartz bottle is about 3.84 cm^2^. Therefore, the total incident energy over the quartz bottle was about 107,495 J in 48 h. A solar-to-hydrogen efficiency of 0.09% was obtained, assuming all incident light was absorbed by optically thick nanoparticle suspension, which was obtained by the following calculation formula: Solar-to-hydrogen efficiency = Output energy of hydrogen generated/Energy of incident light = 93/107495 = 0.09% [1,27].

## 3. Results and Discussion

**Figure 1:**
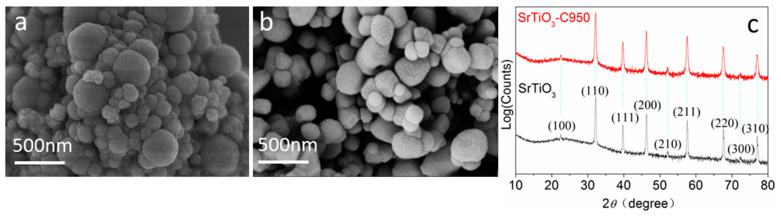
Morphology and Structure characterizations of SrTiO_3_. (**a**), Scanning Electron Microscope (SEM) image of SrTiO_3_; (**b**), SEM of SrTiO_3_-C950; (**c**), XRD of SrTiO_3_ and SrTiO_3_-C950.

Irregular spherical SrTiO_3_ particles with diameters of 100–300 nm were synthesized by a hydrothermal method (Figure 1a) [26]. The carbon reduction process was performed under different calcination temperatures and retention time. It was found that the samples obtained under calcination temperature of 950 °C and a retention time of 3 h under N_2_ atmosphere (marked as SrTiO_3_-C950) and showed the highest photocatalytic activity. Compared with uncalcined SrTiO_3_ particles, the as-prepared SrTiO_3_-C950 particles showed larger particle size (Figure 1b) because of the Ostwald ripening under a high temperature, which was further determined by a laser particle size analyzer (Figure S1). The as-prepared SrTiO_3_ calcined at 950 °C for 3 h with a heating rate of 5 °C/min under air atmosphere was used as a reference sample. As shown in Figure 1c, the X-ray diffraction (XRD) peaks at 22.78, 32.42, 39.98, 46.48, 52.35, 57.79, 67.80, 72.54 and 77.18 can be attributed to the Miller indices of (100), (110), (111), (200), (210), (211), (220), (300) and (310), which can be indexed to the standard cubic perovskite structure (space group: Pm3m) of SrTiO_3_ (JPCDS No. 35–734) without any crystalline by-product. There is no obvious difference in XRD patterns of SrTiO_3_-C950 and the reference SrTiO_3_. This result indicates that the overall crystal structure of SrTiO_3_ is not fundamentally changed under high temperature reduction treatment. No obvious diffraction peaks of graphite carbon are observed due to its amorphous phase or low residue.

**Figure 2:**
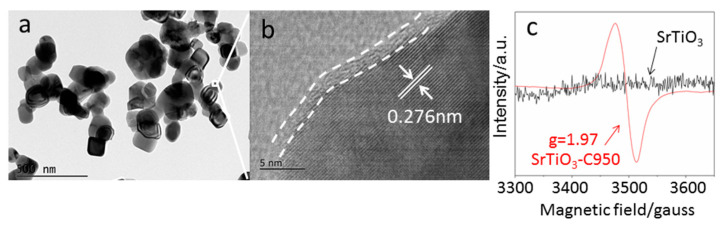
Transmission Electron Microscope (TEM) image (**a**), High Resolution Transmission Electron Microscope (HRTEM) image (**b**) and Electron Paramagnetic Resonance (EPR) image (**c**) of SrTiO_3_-C950.

As shown in Figure 2b, a highly disordered layer with a thickness of 2.0 nm was formed on the surface of SrTiO_3_ after carbonization. It is ascribed to the presence of a large number of oxygen vacancies on the surface of SrTiO_3_ particles caused by the reduction treatment under a high temperature [28], which is further confirmed by EPR and X-ray photoelectron spectroscopy (XPS) analysis. The produced surface oxygen vacancies will act as an electron-trapping center, showing EPR signals. Therefore, EPR is one of the most powerful methods to identify the presence of oxygen vacancies in the solid materials [29]. The low-field signal with g-factor (g is the spectroscopic splitting factor in the case of a free electron) close to the free-electron value (g = 2.0023) is generally attributed to an unpaired electron trapped on an oxygen vacancy site. If the g-value of the light-induced EPR signal is smaller than that of a free electron, 2.0023, the signal is due to the trapped electron. Herein, as shown in Figure 2c, the EPR signal of SrTiO_3_-C950 with a g-value of 1.97 is due to the electrons captured by the oxygen vacancies. As a comparison, there are no EPR signals at the same position observed for SrTiO_3_.

**Figure 3:**
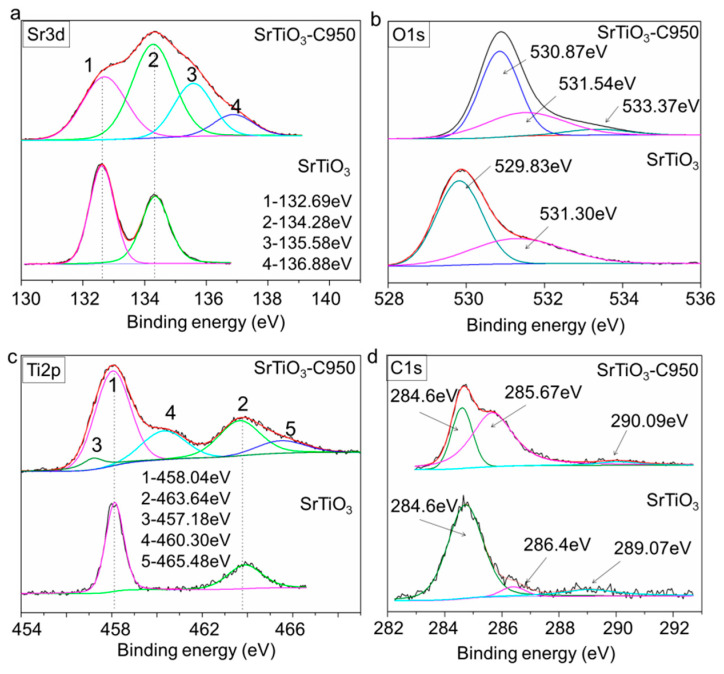
XPS spectra of Sr3d (**a**), O1s (**b**), Ti2p (**c**) and C1s (**d**) of SrTiO_3_ and SrTiO_3_-C950.

The chemical states of Sr, Ti, O and C in SrTiO_3_ and SrTiO_3_-C950 were determined by XPS spectra (Figure S2). As shown in Figure 3a–d, compared with SrTiO_3_, SrTiO_3_-C950 shows distinctly different XPS peaks. The Sr^2+^ 3d5/2 peak centered at 135.58 eV, 136.88 eV and the O1s peak centered at 530.87 eV ascribed to the SrO crystal phase and are observed on SrTiO_3_-C950 (Figure 3a,b) [30]. The as-prepared SrTiO_3_-C950 samples showed the same two Ti2p peaks centered at 458.04 eV (Peak 1) and 463.64 eV (Peak 2) with the referenced SrTiO_3_. However, three reduced titanium ion XPS peaks centered at 457.18 eV (Peak 3), 460.3 eV (Peak 4) and 465.48 eV (Peak 5) were observed for SrTiO_3_-C950, which could be ascribed to the low valence state titanium of nonstoichiometric TiO_2-x_ (0 < X < 2) species, mainly including Ti^2+^ 2p1/2 of TiO (Figure 3c) [31,32], which are quite different from the titanium ion XPS peaks of the SrTiO_3_. The new appearance of SrO and reduced Ti species indicates that there is a large number of oxygen vacancies existing in the surface layer of SrTiO_3_-C950 [33]. Meanwhile, it preliminary indicates that the surface of SrTiO_3_-C950 may be mainly composed of SrO and TiO species layers [34]. The SrO surface layers of SrTiO_3_ was thought to have higher water adsorption dissociation ability [35], which may be more helpful for the photocatalytic water splitting. Because of the existence of carbon standard materials for the XPS test, both the pristine SrTiO_3_ and SrTiO_3_-C950 show obvious carbon peaks (Figure 3d). However, there are significant differences between them on the XPS C1s peaks. For the SrTiO_3_-C950, a C1s peak centered at 285.67 eV ascribed to β-carbon connected with the oxygenic groups on the alkyl chain is believed to originate from carbon reductant [36]. It indicates that there is still a small amount of residual carbon on the surface of strontium titanate. There are no C1s peaks attributed to TiC observed for SrTiO_3_-C950 and pristine SrTiO_3_ [37], indicating that carbon atoms are not doped into a strontium titanate lattice during the reduction process.

**Figure 4:**
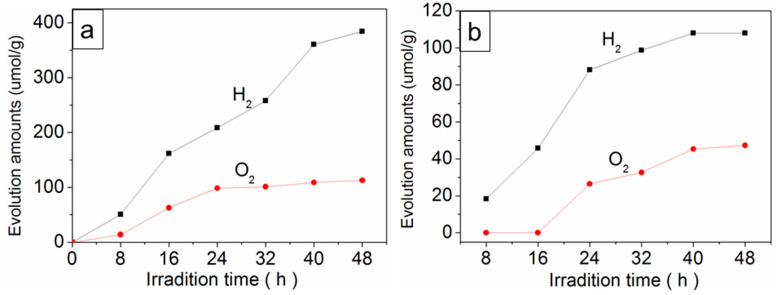
Photocatalytic activity of SrTiO_3_-C950 under full-spectrum light irradiation (**a**) or visible light irradiation (**b**) without sacrificial reagent. No photocatalytic water splitting activity was observed for pristine SrTiO_3_ and SrTiO_3_-C300 under the same conditions.

The photocatalytic activity of SrTiO_3_-C950 and pristine SrTiO_3_ was evaluated by photocatalytic water splitting under Xe lamp light irradiation with or without light cutoff filters (λ > 420 nm) (Figure 4). Compared with pristine SrTiO_3_, the as-prepared SrTiO_3_-C950 shows an obvious photocatalytic water splitting capability with a solar-to-hydrogen (STH) conversion efficiency of 0.09% without any sacrificial reagent. Under the optimal photocatalytic reaction conditions, the average H_2_ evolution rate and O_2_ evolution rate of SrTiO_3_-C950 is 20.6 umol/g×h and 7.5 umol/g∙h under Xe lamp light irradiation, respectively (Figure 4a). Under the visible light irradiation, the average H_2_ evolution rate and O_2_ evolution rate of SrTiO_3_-C950 are 2.3 µmol/g×h and 1.0 µmol/g∙h, respectively (Figure 4b). There is no noticeable difference in the photocatalytic activity of SrTiO_3_-C950 under different pH conditions. In this work, the evolution rates ratio of O_2_ and H_2_ are 0.7:2, which is lower than that of the theoretical stoichiometrically ratio 1:2 of water splitting. This phenomenon has been observed in many water splitting systems, which is due to the produced O_2_ or intermediate oxygen species being readily absorbed by the metal elements to form steady peroxide complexes [38,39].

Although pristine SrTiO_3_ has suitable energy band structure for the water redox potential levels [11], it cannot decompose water into H_2_ and O_2_ simultaneously because of rapid recombination of photogenerated carriers or fast backward reaction. According to the previous reports, it is proposed that the residual carbon on the surface of SrTiO_3_-C950 is favorable for improving the photocatalytic efficiency [40]. However, a referenced SrTiO_3_ modified with carbon calcinated under 300 °C temperatures without reduction treatment, showed no photoactivity on overall water splitting. Therefore, herein, the photocatalytic overall water splitting ability of the as-prepared SrTiO_3_-C950 was suggested to be owed to the surface oxygen vacancies that can make suitable energy levels for visible light response and improve the separation and transfer efficiency of photogenerated carriers [41].

**Figure 5:**
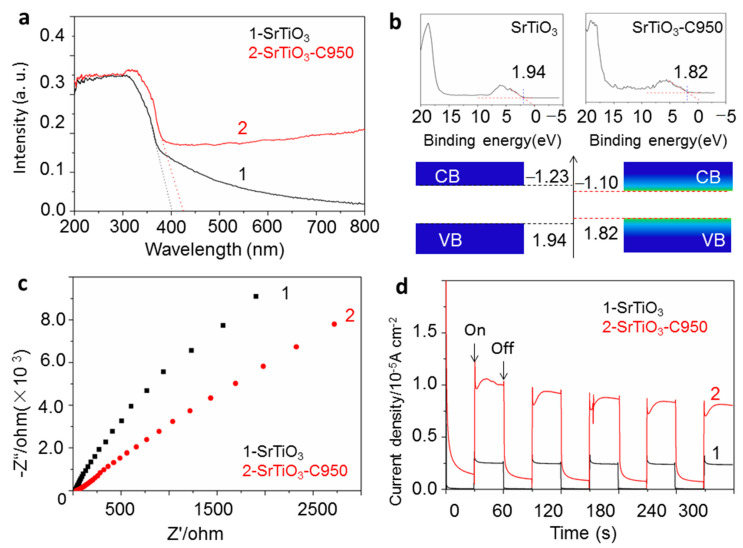
Ultraviolet–visible diffuse reflectance spectrum (UV-Vis DRS) (**a**), XPS Valence Band Spectrum and Energy band structure of SrTiO_3_ (left) and SrTiO_3_-C950 (right) (**b**), Electrochemical impedance spectroscopy (**c**) and Photocurrent density (**d**) of SrTiO_3_-C950 and SrTiO_3_.

As shown in Figure 5a, compared with SrTiO_3_, the absorption wavelength of SrTiO_3_-C950 shows an obvious red shift to the visible light region. Estimated from the UV-V is the DRS absorption tail, the band gap of SrTiO_3_-C950 is 2.92 eV, which is significantly smaller than that of SrTiO_3_ with 3.10 eV. It is due to the formation of the mid-gap states between the valence band and the conduction band, which is attributed to surface oxygen vacancies [19]. These mid-gap states can form a continuum extending to and overlapping with the conduction band edges in a result of narrowing the band gap and expanding the solar absorbance range to lower energy waves. As shown in Figure 5b, obtained from the XPS valence band spectrum (VBXPS), the valence band edge of pristine SrTiO_3_ is localized at 1.94 eV below the Fermi level. Then, it is calculated that the conduction band minimum would localize at about −1.16 eV. The valence band edge of SrTiO_3_-C950 expands to 1.82 eV. In the same way, combined with the band gap of 2.92 eV from optical measurements, the conduction band minimum edge of SrTiO_3_-C950 expands to −1.10 eV. Therefore, the formed energy band structure of SrTiO_3_-C950 is suitable for photocatalytic water splitting under visible light irradiation.

In addition, because of the presence of free electrons bounded loosely in the oxygen vacancies [42,43,44], the surface electric conductivity of SrTiO_3_-C950 will be improved, as a result of improving the carriers’ transfer efficiency, which is also helpful for improving the photocatalytic activity. The electrochemical impedance spectroscopy (Nyquist plot) was used to determine the charge transfer ability. The pristine SrTiO_3_ and SrTiO_3_-C950 all show a classical semicircular Nyquist diagram (Figure 5c). The arc radius of SrTiO_3_-C950 is obviously smaller than that of SrTiO_3_, which indicates that the photogenerated charges in SrTiO_3_-C950 would suffer less resistance than that of SrTiO_3_ and obtain a higher transfer rate. The photocurrent density of SrTiO_3_-C950 was much higher than that of pristine SrTiO_3_ under Xe lamp light irradiation (Figure 5d), which indicates that the charge separation in SrTiO_3_-C950 has been enhanced remarkably. Herein, the photocurrent density of SrTiO_3_-C950 shows a slow decrease with the following cycles, which may be due to the destruction of surface oxygen vacancies under the presence of external electric fields during a photocurrent test. It is proposed that, besides the aforementioned two factors, the higher charge separation efficiency is ascribed to the temporary storage capacity of surface oxygen vacancies for photogenerated carriers [21,45]. Moreover, according to the previous reports, the formed surface oxygen vacancies and SrO species are proposed to be more favorable for the adsorption and decomposition of water molecules [30].

## 4. Conclusions

In summary, a SrTiO_3_ material with surface oxygen vacancies was synthesized by carbon reduction under a high temperature. The as-prepared SrTiO_3_-C950 was successfully applied in photocatalytic water splitting research under visible light irradiation without any sacrificial reagent for the first time. The photocatalytic overall water splitting ability of the as-prepared SrTiO_3_-C950 is attributed to the surface oxygen vacancies that can make suitable energy levels for a visible light response, improve the separation and transfer efficiency of photogenerated carriers. In addition, according to the previous reports, it is proposed that the formed surface oxygen and SrO species is proposed to be more favorable for the adsorption and decomposition of water molecular. This work provided a strategy to design efficient photocatalysts by constructing oxygen vacancies.

## Supplementary Materials

The following are available online at https://www.mdpi.com/2079-4991/10/12/2572/s1. Figure S1: Particle size distribution of SrTiO_3_ before (a) and after (b) carbon reduction process under 950 °C. Figure S2: Survey XPS spectra of SrTiO_3_ (a) and SrTiO_3_-C950 (b).
