# Supplementary material for: Photocatalytic Overall Water Splitting by SrTiO3 with Surface Oxygen Vacancies
Source: Nanomaterials (Basel). 2020 Dec 21;10(12):2572. doi: 10.3390/nano10122572 (PMC7767457; doi:10.3390/nano10122572)
Supplement: Supplementary file 1 [file nanomaterials-10-02572-s001.pdf]

nanomaterials

Supplementary Material

Photocatalytic Overall Water Splitting by $SrTiO_3$ with Surface Oxygen Vacancies

Yanfei Fan, Yan Liu, Hongyu Cui, Wen Wang, Qiaoyan Shang, Xifeng Shi, Guanwei Cui * and Bo Tang

College of Chemistry, Chemical Engineering and Materials Science, Collaborative Innovation Center of Functionalized Probes for Chemical Imaging in Universities of Shandong, Key Laboratory of Molecular and Nano Probes, Ministry of Education, Shandong Normal University, Jinan 250014, China; yffan810@163.com (Y.F.); ly2017020911@163.com (Y.L.); liuliucuicui123@163.com (H.C.); ww19862550729@163.com (W.W.); qiaoyanshang@sdnu.edu.cn (Q.S.); sxf0716@163.com (X.S.); tangb@sdnu.edu.cn (B.T.)

* Correspondence: cuiguanwei@sdnu.edu.cn

1. Physical Characterization

A HITACHI SU8010 scanning electron microscope equipped with a field-emission gun operated at 5.0 kV was used to characterize the morphology of the as-obtained product. The size distribution of $SrTiO_3$ and $SrTiO_3$-C950 particles was determined by Malvern laser particle size analyzer (Malvern Nano-ZS90). High-resolution transmission electron microscopy (HRTEM) was performed on a JEM-2100F instrument at an accelerating voltage of 200 kV. The powder X-ray diffraction patterns (XRD) were collected at room temperature using a scan speed of 0.1 s/step on a Bruker D8 Advance Diffractometer (40 kV, 40 mA) with Cu Kα radiation (1.5418 Å). X-ray photoelectron spectroscopy (XPS) was carried out on Thermo Scientific Escalab 250Xi with Al Kα as the excitation source. The UV/Vis diffuse reflection spectra were taken on a Shimadzu UV-2550 spectrophotometer with an integrated sphere attachment, and $BaSO_4$ was used as the reference sample. Electron paramagnetic resonance (EPR) signals were recorded on a Bruker A300-10/12/S-LC spectrometer with a frequency of 9.87 GHz at room temperature.

2. Supplementary Figures

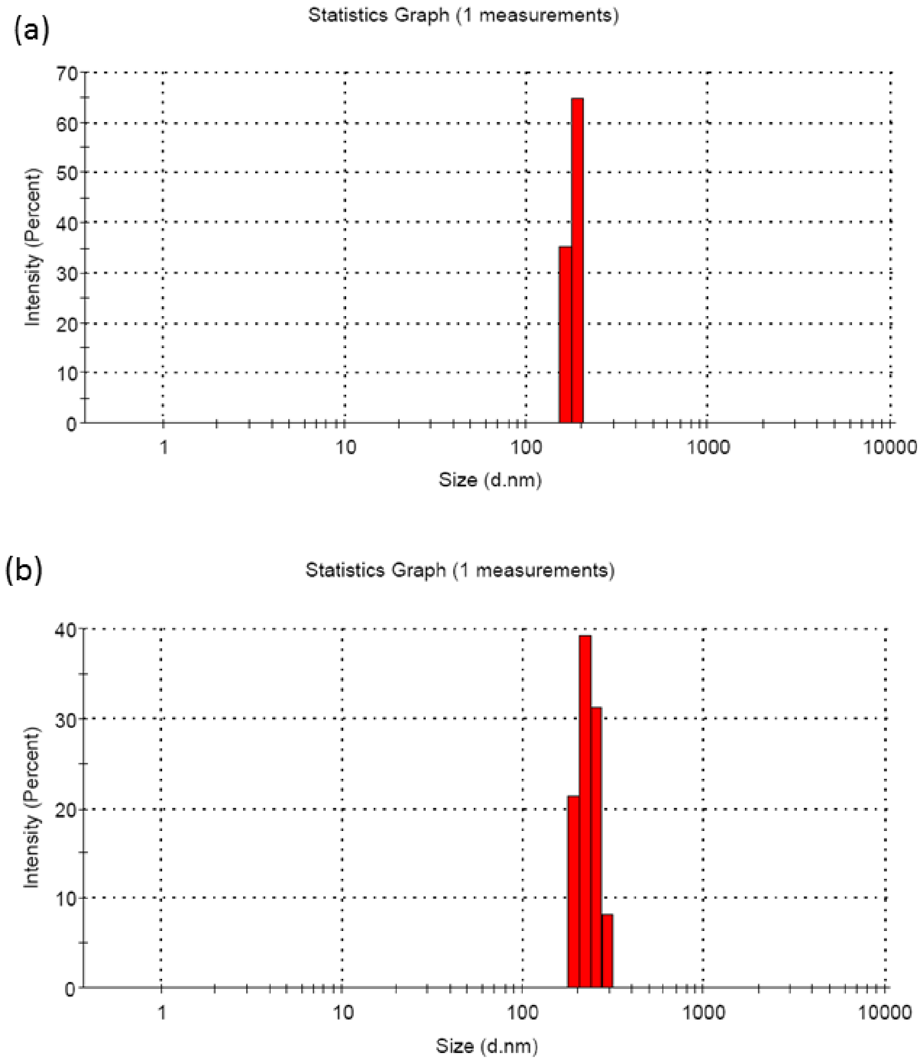

Figure S1. Particle size distribution of $SrTiO_3$ before (**a**) and after (**b**) carbon reduction process under 950 °C

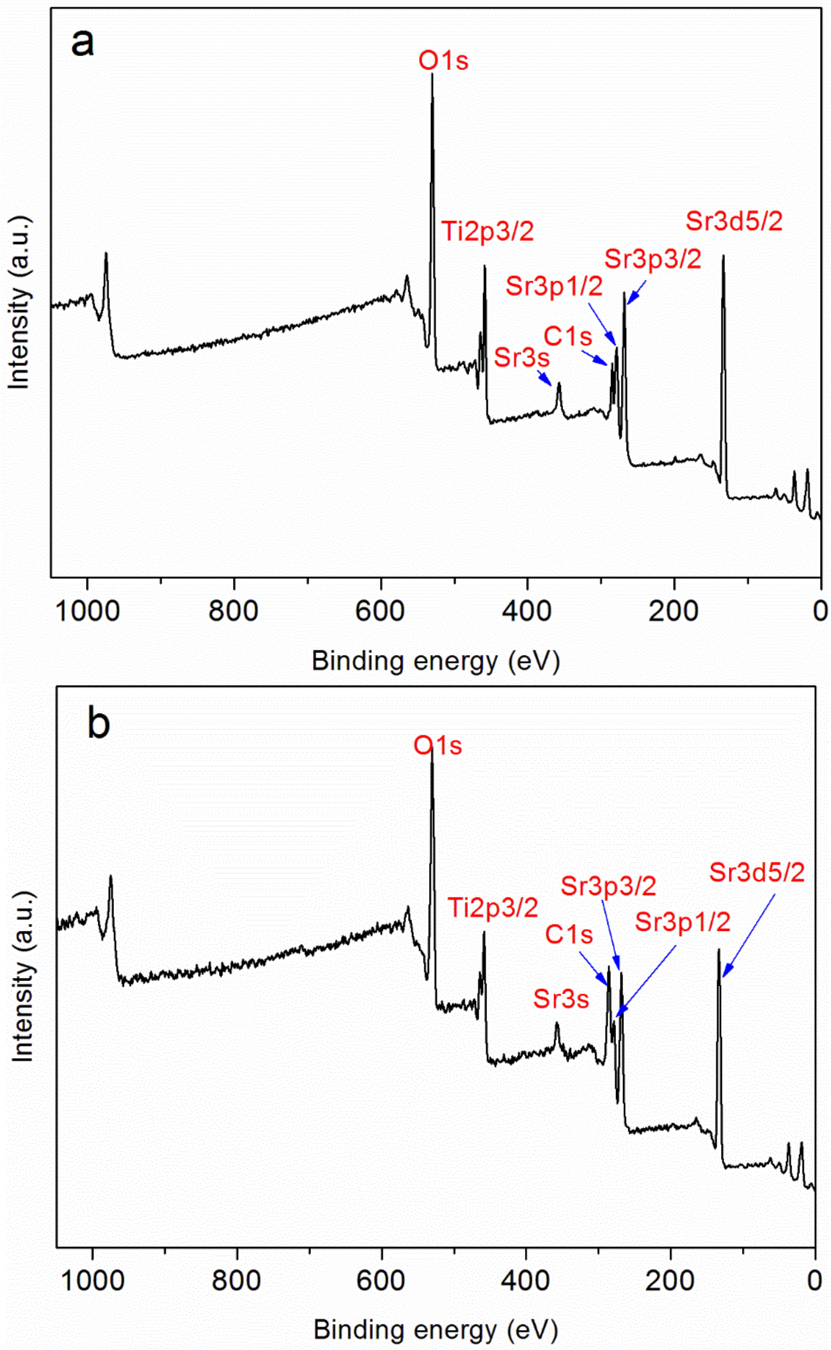

Figure S2. Survey XPS spectra of $SrTiO_3$ (**a**) and $SrTiO_3$-C950 (**b**).
